# Antifungal Effect of Long Noncoding RNA 9708-1 in the Vulvovaginal Candidiasis Murine Model

**DOI:** 10.1007/s11046-021-00530-8

**Published:** 2021-02-15

**Authors:** Ying Wu, Lisha Jiang, Lingling Zhang, Xia Liu, Lina Yan, Ting Luan, Can Rui, Zhiyuan Mao, Chong Fan, Yu Liu, Ping Li, Xin Zeng

**Affiliations:** 1 Women’s Hospital of Nanjing Medical University, Nanjing Maternity and Child Health Care Hospital, Nanjing Medical University, Nanjing, 210004 Jiangsu People’s Republic of China; 2 The Second Affiliated Hospital of Medical University of Anhui, Hefei, 230601 Anhui People’s Republic of China; 3 Jiangsu Taizhou People’s Hospital, Taizhou, 225300 Jiangsu People’s Republic of China; 4 Department of Anatomy, Nanjing Medical University, Histology, and Embryology, Nanjing, 210029 Jiangsu People’s Republic of China

**Keywords:** Vulvovaginal candidiasis, lncRNA 9708-1, Focal adhesion kinase, Inflammation

## Abstract

Vulvovaginal candidiasis (VVC) caused by *Candida spp.* affects 70–75% of women at least once during their lives. We aim to elucidate the potential mechanism of VVC and investigate the therapeutic effects of long noncoding RNA 9708-1. Female BALB/c mice were randomized to four treatment groups, including the blank control group, VVC control group, vehicle control group and lncRNA 9708-1-overexpressed group. Mice were euthanized on Day 4, Day 7 and Day 14 after treatment. Colony-forming unit (CFU) was measured, and the inflammation was detected by hematoxylin and eosin (H&E). Gene and protein expression levels of lncRNA 9708-1 and FAK were determined by real-time PCR, Western blot and immunohistochemistry. The overexpression of lncRNA 9708-1 significantly decreased the fungal load from Day 4 to 7. H&E staining indicated that the impaired histological profiles were improved in lncRNA 9708-1-overexpressed group. LncRNA 9708-1 led to a significant increase in FAK level of vagina tissue which is expressed mainly in epithelial basal layer. This study suggests that lncRNA 9708-1 played a protective role on murine experimental VVC by upregulating the expression levels of FAK.

**Supplementary Information:**

The online version of this article (10.1007/s11046-021-00530-8)

## Introduction

*Candida albicans* (*C. albicans*) which colonizes 30–70% of healthy individuals does not cause significant disease under normal circumstances [1]. Reports indicated the genital infection caused by *Candida spp.* affects 70–75% of women at least once during their lives and 40–50% of women would experience a recurrence [2]. The clinical symptom of vulvovaginal candidiasis (VVC) was copious white vaginal discharge with large numbers of germinated yeast cells, or little discharge but severe pruritus [3]. Most widely used diagnostic method is microscopic examination of vaginal secretions. There is no reliable and clinically useful antigen detection technique available for VVC diagnosis. Antifungal therapy is most widely clinical treatment for VVC, such as miconazole, clotrimazole, and so on. The treatment panel lasts from single-dose therapy to 7 days or even 14 days. RVVC was reported increasing infection of HIV [4], HPV [5] and pelvic inflammatory disease (PID) [6].

*Candida albicans* is the most common infectious yeast strain among *Candida spp.*. They always colonize skin, gastrointestinal and reproductive tracts [7]. The life cycle, genome structure, dynamics, their links to cell biology and adaptation to environmental challenges of *C. albicans* are unique. The features include cell reproduction cycle, chromosome hallmarks, genome, loss of heterozygosity and links between DNA alterations and cell morphology [8]. *C. albicans* invades in tissue via adhesion to the epithelium, epithelial penetration, invasion by hyphae, vascular dissemination, endothelial colonization and penetration during disseminated disease. In the initiation step, the cells propagate to form microcolonies, and germ tubes form to yield hyphae. *C. albicans* biofilms consist of two main kinds of cells: small oval yeast-form cells (also called blastospores) and long tubular hyphal cells. In the adherence step, yeast-form cells adhere to the substrate.

Focal adhesion kinase (FAK) is a nonreceptor tyrosine kinase best known in adhesion mediated signaling. It is able to mediate cell attachment, and dephosphorylation takes place when cells are detached. It also plays an important role in cell migration and apoptosis [9]. In the early interaction between the oral keratinocytes and *C. albicans*, extracellular regulated protein kinases (ERK)-FAK signaling cascades were detected enhancing cell migration [10]. Cell migration is closely associated with disease progression which is regulated by complex pathways [11]. Cells lacking the tyrosine kinases FAK or Src have more and larger adhesions and migrate poorly [12]. Research also showed differential expression of adhesins and secreted products, specific to the infection site [13].

With the development of transcriptome sequencing, more and more noncoding RNAs (ncRNAs) came into sight in recent decades. Although they are nonprotein-coding RNAs, they do play an important role in posttranscriptional and transcriptional regulation in eukaryotes. Those ncRNAs longer than 200 nucleotide are characterized as long noncoding RNAs (lncRNAs) [14]. LncRNAs were most widely studied in oncology with expanding research on infection and immunology. It was suggested lncRNAs could modulate host innate immunity during influenza A virus infection [15] and promote human papillomavirus (HPV)-related tumor’s sensitivity to radiotherapy [16]. Studies of lncRNAs on fungi were increasing in recent years, such as expression change in several lncRNAs [17], promotion of microbial clearance [18], regulation of fungi’s sexual sporulation and subsequent meiotic divisions [19], budding yeast [20], promotion of cellulase expression [21], control of chromatin structure [22], and so on. There are several researches on lncRNAs regulating cancer and inflammation diseases such as pancreatitis through FAK signaling pathway [23, 24]. However, mechanism on lncRNAs regulating fungi’s infection and host immunity is still vague. There are a few researches of noncoding RNAs’ (ncRNA) involved in *Candida albicans* immune and inflammatory responses, such as miR-21, miR-146, miR-132, miR-155 and the let-7 family members [25]. No report was found on how lncRNAs play roles in *C. albicans* infection or vulvovaginal candidosis.

In our previous study, we found that lncRNA 9708-1 (located on chr8:141,05,290–141,060,596 ChiPBase) is expressed at significantly low levels in the vaginal epithelial tissue of menopausal women using a DNA chip assay. Cytological experiment indicated overexpression of lncRNA 9708-1 might increase cell migration and decrease cellular adhesion and FAK was might be the target gene of this lncRNA. Colonization of the vagina requires yeast adherence to vaginal epithelial cells. *C. albicans* gets to the vaginal epithelium mainly through adjacent perianal area via lncRNA 97,089–1/FAK signaling pathway [26]. The present study aimed to characterize the functions of lncRNA 9708-1 in *C. albicans*-induced vaginal infection, and to decipher the mechanism of lncRNA 9708-1 action through FAK signaling pathway. The signaling pathways of adherence and migration of *C. albicans* to the host cells are poorly understood. The present research focuses on general events of vaginal mucosa infection. It might also provide potential common intervention targets for complicated vaginal infection, which would be a revolutionary innovation. Our research should contribute to investigations on the potential regulation of vaginal infection and to devising new methods for treating *C. albicans* vaginal infection, especially the recurrence. It might also shed a new light to developing innovative vaccines for fungal infections.

## Method

### ***Candida albicans*** Culture

*C. albicans* strain ATCC 64,548 was provided by Microbiological Lab of Nanjing Drum Tower Hospital. The strain was recovered and cultivated to the third generation in Sabouraud dextrose agar (SDA) at 35 °C for 72 h. The colonies were harvested, and the final cell concentration was adjusted to 1 × 10^6^/ml in sterile PBS.

### Plasmid Construction and Adenovirus Packaging

The lncRNA-9708–1 overexpression vector, control vector as well as the lncRNA-9708–1 overexpression and control adenovirus particles were constructed and packaged by Shanghai GeneChem Co., LTD. (GeneChem, Shanghai, China). Briefly, the 962-bp-length lncRNA-9708–1 (human, LOC105375784) transcript was amplified and cloned into GV315 adenovirus vector, which also encodes the enhanced green fluorescent protein. To prepare adenovirus particles, the adenovirus vector GV315, the helper plasmid PBHG plasmid were cotransfected into HEK293 cells (ATCC, cat#CRL-1573) according to the manufacturer's recommended protocol. And the adenovirus particles were harvested after virus amplification and purification.

### Murine Model

Female BALB/c mice (6–8 weeks, 18–21 g) purchased from the Model Animal Research Center of Nanjing University were maintained with a 12 h light/dark cycle and allowed free access to food and water supplies. Animal experiments were performed in accordance with the Institutional Guidelines for Animal Care framed by the Nanjing Medical University, China. Mice were injected intraperitoneally with 0.1 mg β-estradiol (Sigma-Aldrich, USA) in 100 mL filter-sterilized sesame oil three days prior to inoculation. Mice were anaesthetized with isoflurane and inoculated vaginally with 20 μL *C. albicans* in sterile PBS, followed by vagina submucosa injection of NC or lncRNA 9708-1-overexpressed adenovirus (10^9^ plaque-forming unit/mouse) into the mouse posterior vaginal wall at 3, 6 and 9 o’clock over 5 s.

### *Antifungal Effects of lncRNA 9708-1 *in vivo

#### Quantification of Vaginal Fungal Burden

Vaginal washes were collected by flushing vaginas with 30 μL sterile PBS using a P200 pipet (Axygen, New York, USA) followed by rinsing into an additional 10 mL PBS in a sterile 1.5 mL Eppendorf tube. *C. albicans* titers were determined from washes by preparing tenfold serial dilutions in PBS (in the anaerobic chamber) and spotting 200 μL of each dilution in quadruplicate onto culturing media on tryptic soy agar (TSA) plates at 37 °C . Colonies were then enumerated and reported as recovered colony-forming units (CFU) per mL of vaginal fluid.

#### Histological Analysis of Vaginal Tissue

Mice were sacrificed at 4th, 7th and 14th days to harvest vagina. The vagina from each mouse was homogenized followed by serial dilution and plating as for vaginal washes. The vagina of each mice was used for histological and immunochemical analyses. Part were fixed in 4% buffered formalin phosphate at room temperature followed by paraffin embedding. Part of sections were analyzed for enhanced green fluorescent protein (EGFP) expression by fluorescence microscopy after treatment with 0.5% Chicago Sky Blue (Sigma, USA) to inhibit the background autofluorescence of the tissue. The remaining vaginal tissue from each mouse was stored at − 80 °C for real-time PCR and Western blot. Cell nuclei were counterstained by DAPI (Sigma, USA). Histological slide preparation and H&E staining were performed by the Servicebio Company. Histological and immunofluorescence analyses were performed on a Zeiss Primo Star microscope. The inflammation of vagina tissue was scored by eye independently by three observers unaware of the estrus phase of mice.

#### Quantification of lncRNA9708-1 and FAK

Total RNA was isolated from murine vaginal tissue using Trizol reagent (Invitrogen, Carlsbad, CA), according to the manufacturer’s protocol. LncRNA 9708-1 was reverse-transcribed to cDNA by using Reverse Transcription Kit (Takara, Japan), and mice β-actin expression was used as the internal control. RT-PCR was performed using a Quant Studio 6 flex real-time PCR system (Thermo Fisher, USA). The 20 µL reaction volume included 100 ng RT product, 10 µL 2◊TaqMan Universal Master Mix II and 1 µL primers and was performed for 2 min at 50 °C, 10 min at 95 °C,15 s at 95 °C and 1 min at 60 °C for 40 cycles followed by the thermal denaturation protocol. All reactions were done in triplicate. The expression of lncRNA 9708-1 was defined based on the threshold cycle (Ct), and relative expression levels were calculated as 2^−△△Ct^ after normalization with reference to expression of β-actin. The primers used for the detection of lncRNA 9708-1 levels in mice vaginal samples were as follows: 5′- GGAACCCAAAACCAAAAG-3′ (forward) and 5′-CCCTGAATGACCAACAAA-3′ (reverse); FAK levels as follows, 5′- CACTGGATCTCGGGCTAGGAT (forward) and 5′- GCCTTATGACGAAATGTTGGG-3′ (reverse); β-actin levels as follows, 5′-GGCTGTATTCCCCTCCATCG -3′(forward), 5′- GGCTGTATTCCCCTCCATCG-3′ (reverse).

#### Analysis of FAK Protein

Total protein was obtained using RIPA buffer with cocktail inhibitors (Cell Signaling Tech, USA). Protein concentration was measured using a BCA kit (Pierce, USA). Equal amounts of protein were separated on a 15% gel and then transferred to 0.22 μm PVDF membranes (AmerSham, USA). The membranes were blocked in 5% bovine serum albumin (BSA) in Tris-buffered saline with Tween 20 buffer (TBST) for 2 h and then incubated overnight at 4 °C with the following primary antibodies: FAK-antibody (1:1000, Cell Signaling Tech, USA) and anti-β-actin (1:1000, Cell Signaling Tech, USA). Then, the membranes were washed three times with TBST and incubated with horseradish peroxidase-conjugated secondary antibody (goat anti-rabbit IgG, 1/5000 or goat anti-mouse IgG, 1:5000, Cell Signaling Tech, USA) for 1 h at room temperature. Blots were developed using a chemiluminescence kit (Pierce, USA) and exposed to X-ray film. The bands on the film were scanned and analyzed with Quality One software (Bio-Rad). Primary antibodies and conditions used to probe blots were rabbit anti-FAK (1:1000; Cell Signaling, USA), mouse anti-β-actin (1:1000; Cell Signaling, USA). Appropriate HRP-conjugated secondary anti-rabbit or anti-mouse antibodies were used (Cell Signaling, USA). The FAK protein expression level was normalized to β-actin expression on the same nitrocellulose membrane.

#### FAK Expression in Mice Vaginal Tissue

For microscopy of FAK, cryosections were immunofluorescently labeled with primary antibodies used for IHC which were FAK Antibody (1:200; Cell Signaling) [27]. Antibody incubations were carried out at 4 °C in a humidified chamber, overnight. Bound antibody was visualized using anti-rabbit (1:50; Cell Signaling, USA) secondary antibody conjugated with fluorescein isothiocyanate. Fluorescently labeled sections were treated with 95% ethanol and then mounted under coverslips with Vectashield mountant containing propidium iodide (Vector Laboratories) before viewing under the microscope. Control sections were processed as above with omission of the primary antibodies. Microscopy was performed using a Zeiss Primo Star microscope.

### Statistical Analysis

All statistical analysis was performed using SPSS 22.0 software. The data are expressed as the mean ± standard deviation (SD). One-way ANOVA was utilized for multiple group comparisons. When significant differences were indicated by ANOVA, intergroup comparisons were made using the student’s *t* test. *P* < 0.05 was considered significant.

## Results

### Experimental Murine VVC Model

**Fig. 1:**
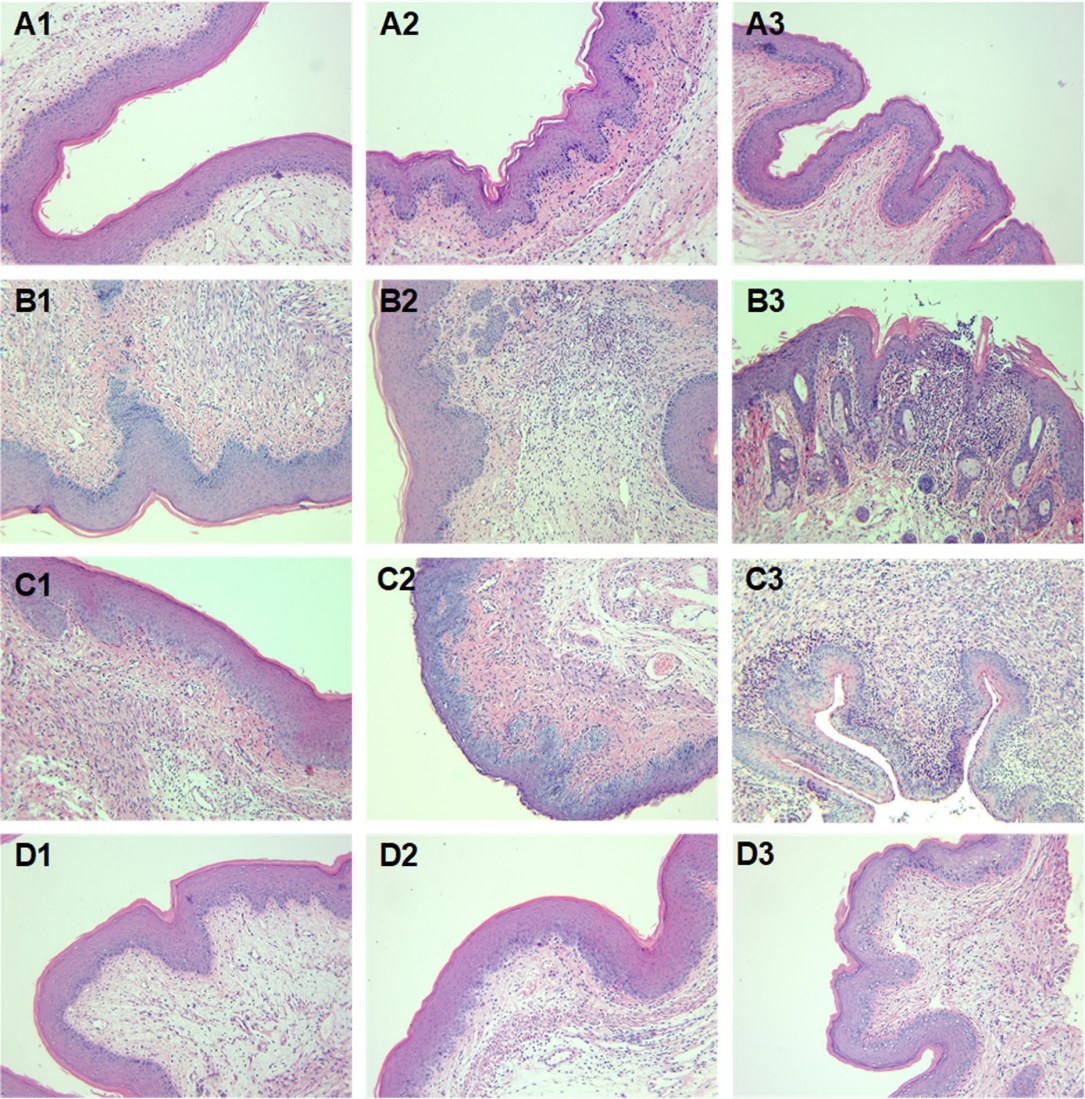
Histologic changes in BALB/c mice after *C. albicans* infection and lncRNA9708-1 overexpression. Histological inflammation was assessed by hematoxylin–eosin (H&E) staining of formalin-fixed, paraffin-embedded vaginal tissue sections. Photomicrographs were taken from submucosal and epithelial areas 4, 7 and 14 days after submucosal adenovirus infection (1 × 10^9^ pfu/mouse). Images are representative of sections (200 ×) from at least three mice. (A1-3) Images of H&E-stained vagina from the blank control groups of mice showed the vaginal epithelium of the mice covered with squamous epithelium and keratinized surface layer. (B1-2) On the 4th and 7th day after C. albicans infection, lymphocytes and neutrophils infiltrated in the interstitium of tissue sections of the mice vaginal wall. (B3) On the 14th day, the mice of *C. albicans* control group presented partial vaginal epithelium deletion with erosion, lymphocytes and neutrophils nest aggregation and infiltration were observed in the inferior stroma. (C1-3) Lymphatic and neutrophils can be seen on the surface of the vaginal tissue, and a large number of lymphocytes and neutrophil infiltration are observed in the lower interstitium. (D1-3) The vaginal epithelium of the lncRNA 9708-1-overexpressed group showed a small amount of lymphocytes and neutrophil infiltration in the interstitium, and the inflammatory response was lighter than that of the vehicle control group

There were no differences in body weight on the 1st, 7th and 14th day in each group. Neither *C. albicans* infection nor lncRNA 9708-1 treatment led to weight loss during the treatment course (Fig. s1). Vulva of *C. albicans* infected mice appeared red by inflammation and thick white discharge. On the 4th day, the vulva of the mice was observed: blank control group was completely normal; the vulva of *C. albicans* control and vehicle control group was red and swollen obviously, and there was a lot of white secretion. Vaginal fungus culture showed a significant increase in *C. albicans*-infected mouse after implantation (Figs. s2a, b and s3). To discover inflammation in the vaginal tissue, H&E staining was performed. The vaginal epithelium of the mice in the blank control group showed covered squamous epithelium and keratinized surface layer (Fig. 1a1–a3). On the 4th and 7th day after *C. albicans* infection, lymphocytes and neutrophils infiltrated in the interstitium of tissue sections of the mice vaginal wall (Fig. 1b1–b2). On the 14th day, the mice of *C. albicans* control group presented partial vaginal epithelium deletion with erosion, and a large number of lymphocytes and neutrophils nest aggregation and infiltration which were observed in the inferior stroma (Fig. 1b3). The pathological manifestations of vaginal epithelium in the vehicle control group were similar to those in the *C. albicans* control group. Lymphatic and neutrophils are seen on the surface of the vaginal tissue, and a large number of lymphocytes and neutrophil infiltration are observed in the lower interstitium (Fig. 1c1–c3). The vaginal epithelium of the lncRNA 9708-1-overexpressed group showed a small amount of lymphocytes and neutrophil infiltration in the interstitial, and the inflammatory response was lighter than that of the vehicle control group (Fig. 1d1–d3).

### C. albicans Infection Increases the Expression of lncRNA9708-1 and FAK in Murine Vagina

**Fig. 2:**
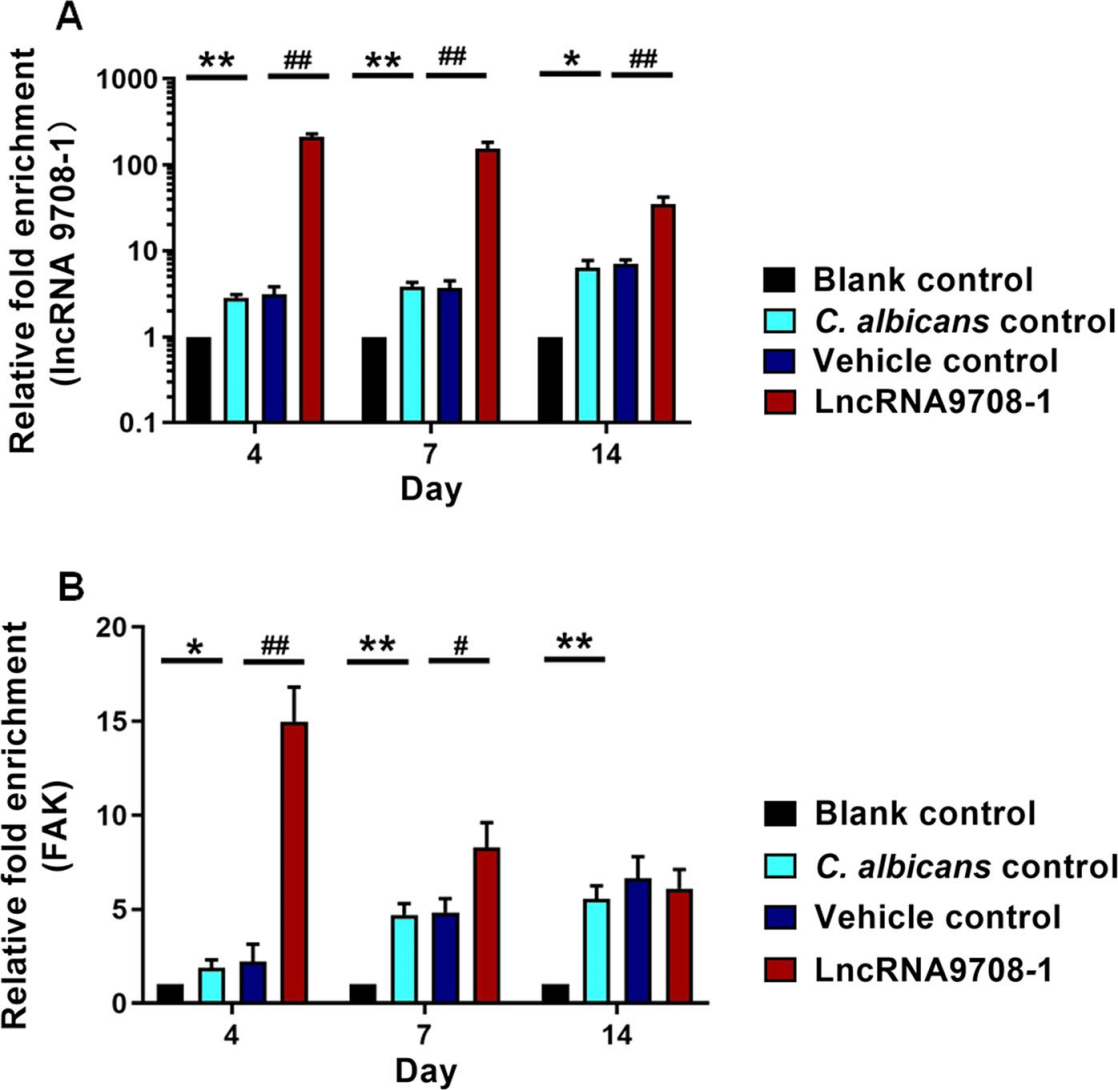
**a** Amount of lncRNA 9708-1 expression in mice. Relative RNA expression levels of lncRNA 9708-1 in vaginal tissues dissected from BALB/c mice (*n* = 3 per group). Tissues were harvested, and RT-qPCR analysis was performed to quantify lncRNA 9708-1 expression levels. *P < 0.05 and **P < 0.01 in *C. albicans*-infected mouse versus control, ^#^*P* < 0.05 and ^##^*P* < 0.01 in lncRNA 9708-1-overexpressed mouse *vs*. control, unpaired *t*-test. Data were presented as mean ± SD. Error bars represent the SD from the mean of three independent experiments. **b** The amount of FAK expression in mice. Relative RNA expression levels of FAK in vaginal tissues dissected from BALB/c mice (*n* = 3 per group). Tissues were harvested and RT-qPCR analysis was performed to quantify FAK expression levels. *P < 0.05 and **P < 0.01 in *C. albicans*-infected mouse versus control, ^#^*P* < 0.05 and ^##^*P* < 0.01 in lncRNA 9708-1-overexpressed mice versus control, unpaired *t*-test. Data were presented as mean ± SD. Error bars represent the SD from the mean of three independent experiments

**Fig. 3:**
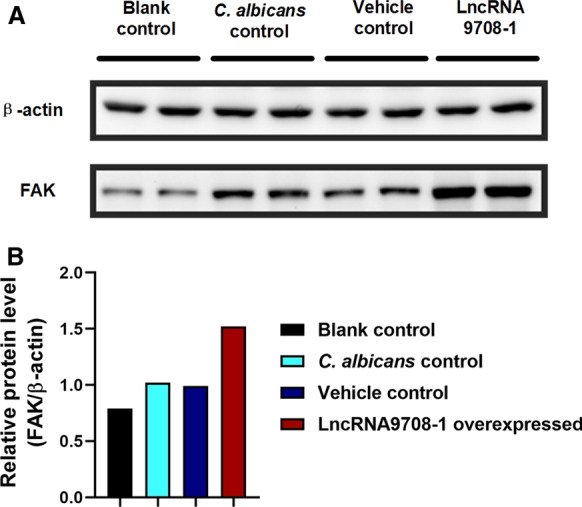
FAK protein expression in mice. **a** Representative Western blots of FAK protein expression in mice vagina. **b** Graph shows increased FAK on 4 days postinjection of lncRNA 9708-1-overexpressed adenovirus compared with vehicle control mice. Western blot analysis showed there was no significantly different FAK expression in *C. albicans*-infected mice with blank control mice (p > 0.05 in *C. albicans*-infected mouse *vs*. control). ^##^*p* < 0.01 in lncRNA 9708-1-overexpressed mouse vs. control, unpaired *t* test. Data were presented as mean ± SD. Error bars represent the SD from the mean of three independent experiments

To uncover the potential molecular mechanism of lncRNA9708-1 on mice VVC, the expression of lncRNA9708-1 and FAK was detected. We compared mice on 4, 7 and 14 days after *C. albicans* infection and examined the mRNA level of lncRNA9708-1 and FAK in their vaginal tissues. The lncRNA9708-1 level increased 2.88-fold on day 4, 3.86-fold on day 7 and 1.27 folds on day 14 versus blank control group. The mRNA of FAK was discovered 1.87 folds higher on day 4, 4.72 folds higher on day 7 and 5.56 folds higher on day 14 expressed than the blank control group (Fig. 1). The expression of FAK protein is significant higher by Western blot (Fig. 2). Immunohistochemical analysis showed FAK expressed in mice vagina in both normal vaginal tissue and vaginosis tissue. Immunohistochemical analysis showed in *C. albicans*-infected vagina FAK was detected higher in the cytoplasm of the basal cells, parabasal cells, vascular endothelial cells and leukocytes than control group (Fig. 3).

Overexpression of lncRNA9708-1 inhibits vaginal inflammation in mice model.

**Fig. 4:**
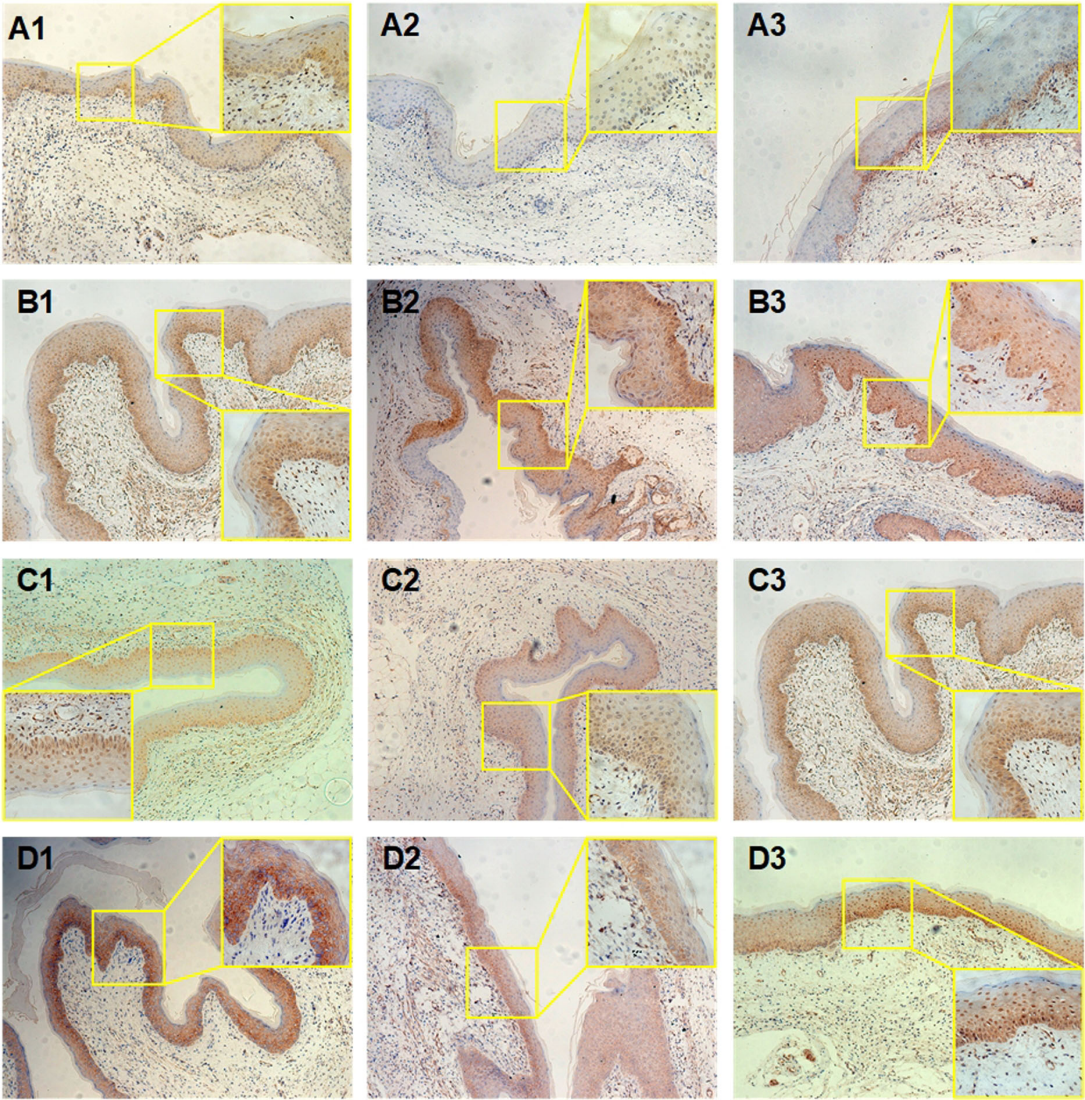
Immunohistochemical detection of FAK proteins in vaginal tissues. Tissue sections were incubated with an antibody against FAK. All immunostains were independently scored by two independent observers. Representative Immunohistochemical photograph of **a** control mice vagina, in which low levels of FAK were expressed in normal vaginal epithelium. **b** FAK expression of *C. albicans*-infected mice vagina markedly increases after 4 days of fungi implantation. **c** FAK expression in *C. albicans*-infected and adenovirus vector control mice. FAK is upregulated in both *C. albicans*-infected mice vagina and *C. albicans*-infected and adenovirus vector control mice compared with control mice. There is no conspicuous difference between these two groups. **d** Vaginal FAK expression in *C. albicans*-infected and lncRNA 9708-1-overexpressed mice is remarkable upregulated compared to *C. albicans*-infected and adenovirus vector control mice

**Fig. 5:**
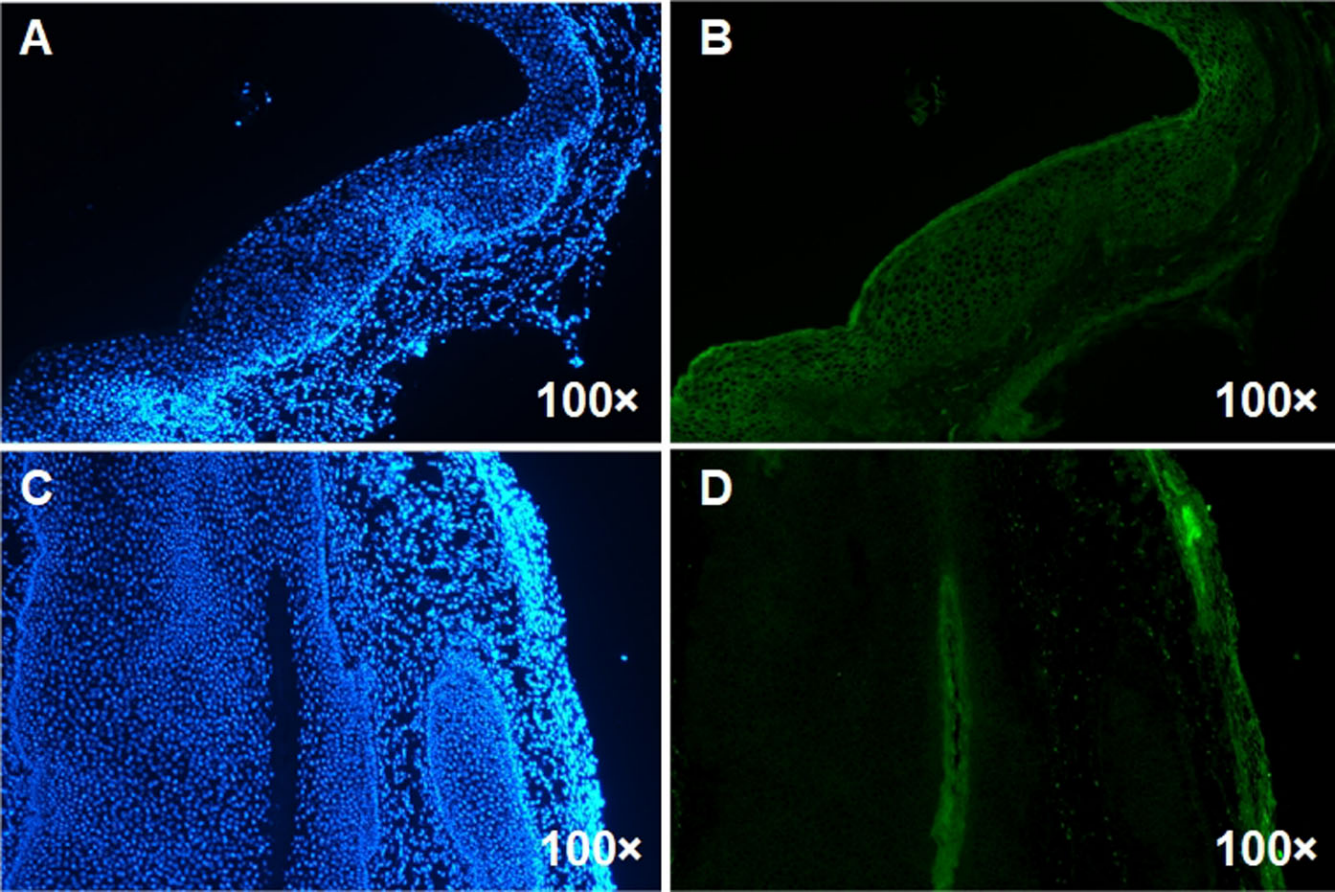
GFP expression in mice vagina. **a** GFP expression in lncRNA 9708-1-overexpressed mouse 4 days post-adenovirus injection. **b** DAPI stain in lncRNA 9708-1-overexpressed mouse 4 days post-adenovirus injection. **c** GFP expression in vehicle control mouse 4 days post-adenovirus injection. **d** DAPI stain in vehicle control mouse 4 days post-adenovirus injection

Mice infected with *C. albicans* were vaginal submucosal injected with lncRNA9708-1-overexpressed adenovirus or control vehicle. After four days, we found that both adenoviruses were shown in the vaginal epithelium by green fluorescent protein (GFP) and 4′,6-diamidino-2-phenylindole (DAPI) staining (Fig. 4). Submucosal injection of lncRNA 9708-1-overexpressed adenovirus which also expressing GFP (AdCAGGFP) shows significant transduction of murine vaginal at 4 days post-injection. We also observed fluorescence on day 7 and day 14 after injection, which gradually faded to undetectable. A representative image of a transverse section of mouse vagina showing GFP transduction is presented. DAPI stain of cell nuclei of the same vagina AZ section is also shown. GFP expression was observed along the vaginal epithelium, with a little interstitial tissue expressing GFP. On the 4th day, the vulva of the mice was observed: vehicle control group were severely red and swollen, and there was a lot of white secretion, while the syndromes of lncRNA 9708-1-overexpressed mice were slight. On 4th, 7th and 14th days, postinjection of lncRNA9708-1, murine vagina tissue was collected. Pathological results showed comparing with the blank control group, the number of inflammatory cells in the vaginal epithelium of *C. albicans* control and vehicle control increased significantly, while the number of inflammatory cells of the lncRNA9708-1-overexpressed group was significantly lower than that of the previous two groups (Fig. 5c–d), and the count of bacteria was significantly reduced (Fig. s2). Real-time PCR showed the lncRNA9708-1 level increased 67.67-fold (4 days), 41.37-fold (7 days); 4.92-fold (14 days); and FAK increased 2.23-fold (4 days); 4.84-fold (7 days); 6.66-fold (14 days) after lncRNA9708-1 overexpressing (Fig. 1). The expression of FAK protein is significantly higher than *C. albicans* vehicle control mice on the 7th day (Fig. 2). FAK expression in mice vaginal was investigated by immunohistochemical analysis. Immunostaining of FAK was seen in the cytoplasm of all cells, particularly in cells located in the uplevel-epithelium cells. (Fig. 3). LncRNA9708-1-overexpressed tissues were stained more intensely than the normal epithelial cells.

## Discussion

VVC is one of the common gynecological infectious diseases in women of reproductive age. The predisposing factors of VVC include genetic factors, hormones, age, sexual activity, the use of antibacterial agents and so on [28]. VVC as an infectious disease elicits immune responses in the host. *Candida albicans* was capable of avoiding adherence to abiotic and host surfaces [29], producing tissue-invading filaments (hyphae) [30], and resistance to antifungal therapy [31]. These serious phenomena require new strategies to prevent or treat VVC. In recent years, a vaccine based on the N-terminus of Als3 protein formulated with alum NDV-3 is reported in protected patients of RVVC from recurrence [32]. TOL-463 was also found effective and safe in treating VVC and reducing recurrence rates, combined with traditional antimicrobials [33]. MicroRNA was discovered to inhibit cell apoptosis and promote cell growth in murine VVC model [34].

It is widely accepted that field planting and invasion are early events in the pathogenesis of *C. albicans* [35]. However, the early molecular events (e.g., migration, adhesion) in response to *C. albicans* challenge are still elusive. Cell migration is closely related to disease progression. *C. albicans* rearranged the cellular actin, hence leading to regulating cell migration [36]. Accumulating evidence indicated that FAK is important for cell migration and adhesion; however, the role of FAK in the regulation of *C. albicans* adherence to extracellular matrix (ECM) and to host cells has been poorly elucidated. FAK which controls cytoskeletal dynamics and cell adhesion by regulating survival and growth signaling may be involved in the primary interaction between the first defense barrier with *C. albicans* [37]. Study also demonstrated that the *C. albicans* attack is capable of enhancing the migration of oral keratinocytes through the activation of ERK/FAK pathway [10].

At present, studies on the relationship between lncRNAs and FAK signaling pathway are mostly on tumors but infectious diseases. There was no research on lncRNA’s function in VVC till today. Zheng’s found lncRNA FAM225A promoted nasopharyngeal carcinogenesis cell proliferation, migration, invasion, tumor growth and metastasis via FAK signaling pathway [38]. LncRNA DBH-AS1 was reported regulating cell motility and invasion via FAK/ERK pathway [39]. LncRNA- ITGB2-AS1 could promote the migration and invasion of breast cancer by activating integrin-related FAK signaling [40]. Our study provides evidence that the lncRNA 9708-1 associated FAK signaling pathway at both the gene and protein levels. To explore the potential mechanism of lncRNA 9708-1 in VVC in vivo, we employed the vaginal candidiasis mouse model. Candida vaginal colonization was detected persisting for weeks in mice [41]. Our present study demonstrated when vagina was attacked by *C. albicans*, the expression of lncRNA 9708-1 which was normally low expressed in vagina tissue upregulated and activated the key pathway in migration FAK, and then the migratory ability of cells increased remarkably. Moreover, the research revealed potential mechanism that FAK signaling plays a role in VVC for the significant reduction of inflammatory response after overexpression of lncRNA 9708-1. Our study suggests a relationship between lncRNA 9708-1 and *C. albicans* infection which may be mediated by the FAK pathway. According to the ChIPBase database analysis, there is an estrogen response element (ERE) TGACCTCAGGTGA at the − 1112 bp upstream of lncRNA 9708-1. The mechanism involved in estrogen regulation of vaginal inflammatory response needs to be further studied. FAK signaling cascades were found involved in the early interaction between the vaginal epithelial cells and *C. albicans* [42]. Our research suggested the overexpression lncRNA 9708-1 upregulated the expression of FAK. It was observed that fungal burden in vagina decreased and the inflammatory response is relived. These phenomena showed that lncRNA 9708-1 has positive effects on VVC.

FAK was found as a mediator of proliferation and survival which regulate apoptosis and survival due to the ECM. MiRNAs were discovered participated in a feedback regulation to the ECM such as miR-29 [43]. FAK was also detected playing an role in cytoskeletal perturbations during virus infection and activation of innate immune signaling [44]. Other studies have shown that the marasmius oreades agglutinin (MOA) reduced FAK phosphorylation to impair cell survival by disturbing disruption of the cytoskeleton [45]. Although there are many studies on FAK, most of them focus on the mechanism of tumorigenesis, but not many studies on infection.

LncRNAs can regulate gene expression by interacting with genomic DNA, RNA, or proteins and regulate a variety of biological processes at the posttranscriptional level. Several lncRNAs have been investigated to have a key role in modulating immunity against microbial components [46]. LncRNAs could function via the regulation of growth and replication of pathogens, via cell-autonomous antimicrobial defense mechanisms or promoting microbial clearance [47]. However, the signaling pathways or cellular events which regulate microbial infection remain poorly understood.

It is widely accepted that many tumors are inextricably linked to infection, such as Hepatitis B virus (HBV) and hepatocellular carcinoma (HCC), Epstein–Barr virus (EBV) with Burkitt’s lymphoma and nasopharyngeal carcinoma, human papillomavirus (HPV) and cervical cancer. The role of lncRNAs in inflammation may be more prospective.

In conclusion, we provide here evidence in that both lncRNA 9708-1 and FAK are activated in murine experimental model of VVC. Our results suggest that sequential activation of lncRNA 9708-1 regulates *C. albicans* vaginal infection through FAK signaling. However, there are still some weaknesses in our study. Since adenovirus concentration attenuates rapidly in vivo in mice, we found it very difficult to perform lncRNA 9708-1 pulldown experiment. We will address these issues in future studies. We believed our research might shed a new light of an attractive target on VVC therapy.

## Supplementary Information

Fig. s1 The body weight of mice. There was no difference in body weight between the four groups at day 0, 4, 7, and 14, unpaired t test. Data were presented as mean ± SD. Error bars represent the SD from the mean of three independent experiments (JPG 114 kb)

Fig. s2 Vaginal bacteria culture after colonization of C. albicans in mice vagina. Vaginal washes were collected by flushing vaginas and pipetting up and down 10x. Colonies were then enumerated and reported as recovered colony-forming units (CFU) per mL of vaginal fluid. Typical AGARs are presented in the figure. (a1-3) Blank control mice. (b1-3) C. albicans vaginal infected mice. (C1-3) C. albicans vaginal infected mice with adenovirus vehicle control. (d1-3) C. albicans vaginal infected mice with lncRNA9708-1 overexpression (JPG 837 kb)

Fig. s3 Vaginal bacteria load after C. albicans implantation. C. albicans titers were determined by enumerating colony-forming units (CFU) in vaginal washes at 4,7, 14 day after infection. Results are meta data from three independent experiments, each with nine mice infection group (JPG 80 kb)
