# Supplementary figures and images for: Antifungal Effect of Long Noncoding RNA 9708-1 in the Vulvovaginal Candidiasis Murine Model
Source: Mycopathologia. 2021 Feb 15;186(2):177–88. doi: 10.1007/s11046-021-00530-8 (PMC8106589; doi:10.1007/s11046-021-00530-8)

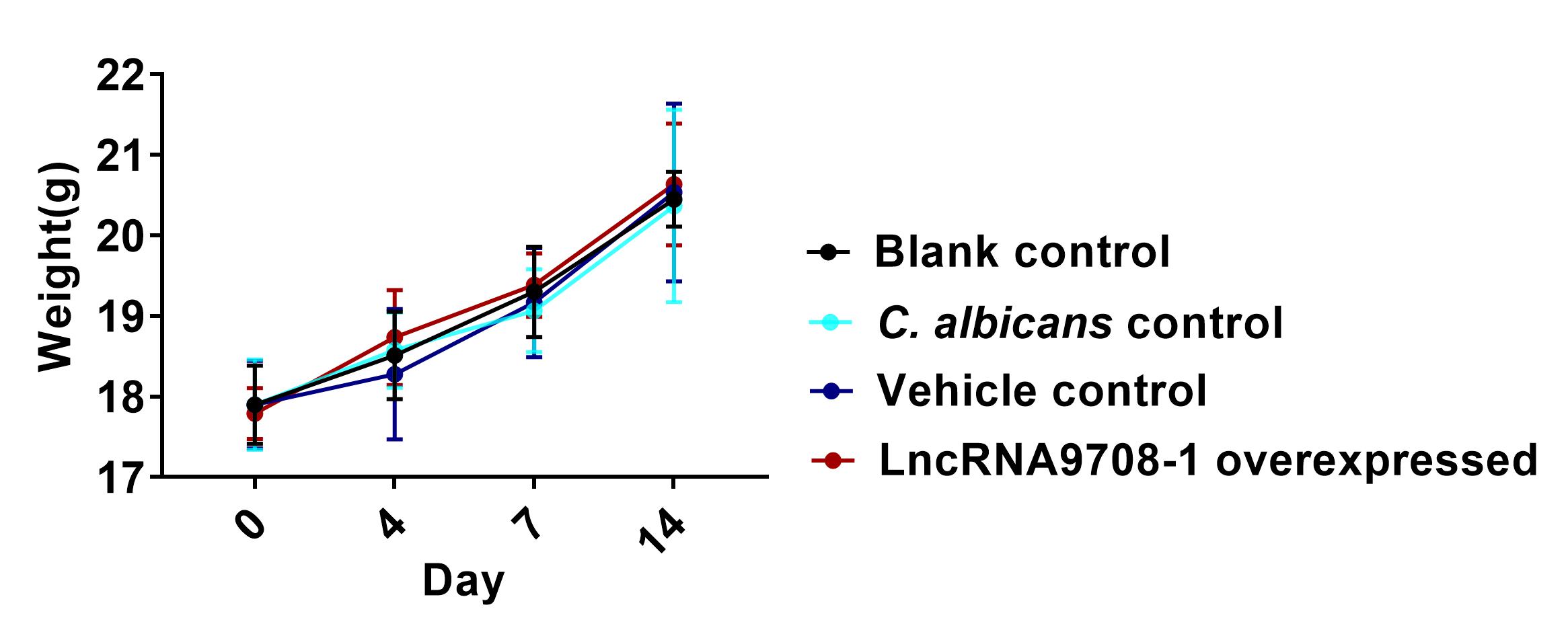

Supplement: Supplementary file 1 — The body weight of mice. There was no difference in body weight between the four groups at day 0, 4, 7, and 14, unpaired t test. Data were presented as mean ± SD. Error bars represent the SD from the mean of three independent experiments (JPG 114 kb) [file 11046_2021_530_MOESM1_ESM.jpg]

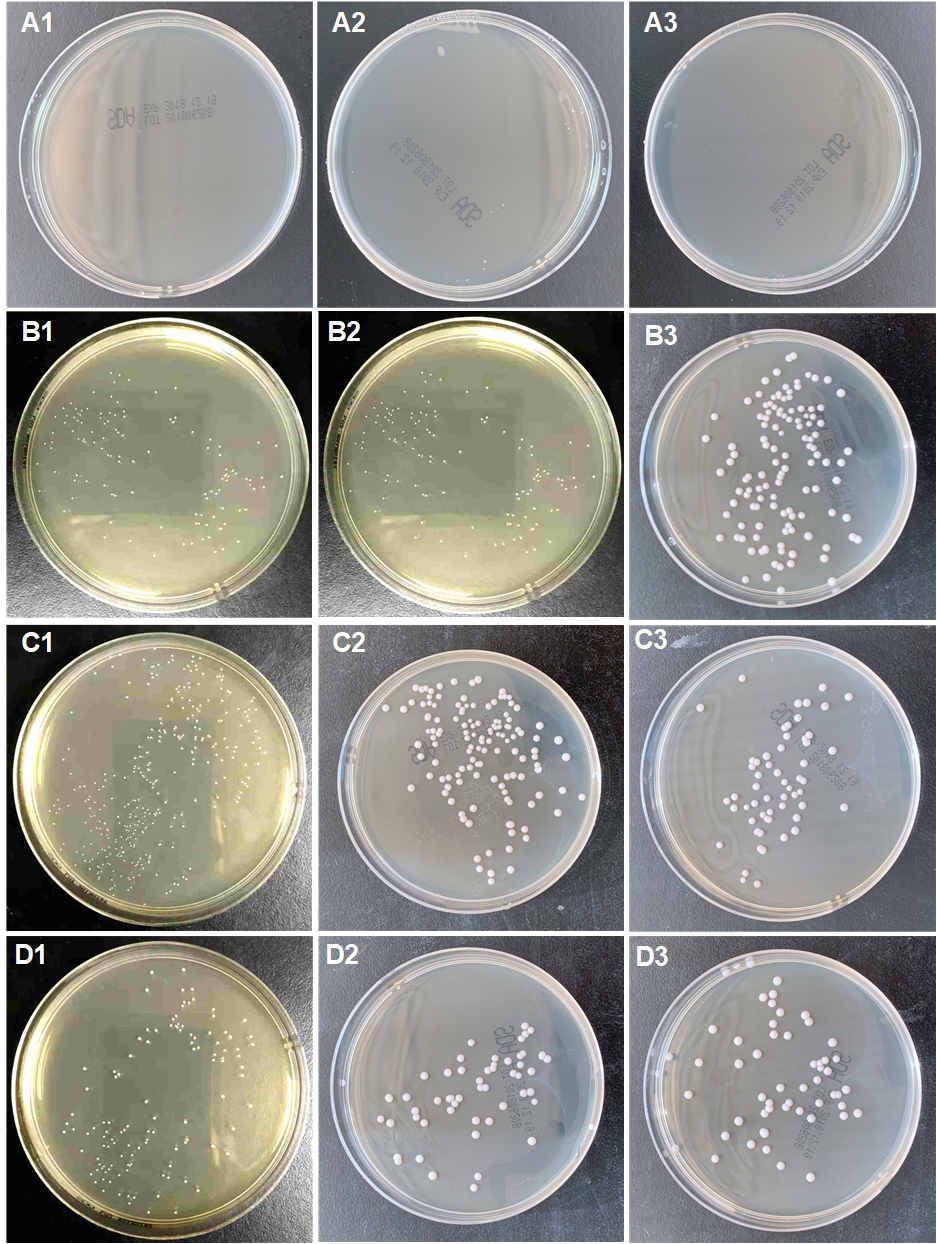

Supplement: Supplementary file 2 — Vaginal bacteria culture after colonization of C. albicans in mice vagina. Vaginal washes were collected by flushing vaginas and pipetting up and down 10x. Colonies were then enumerated and reported as recovered colony-forming units (CFU) per mL of vaginal fluid. Typical AGARs are presented in the figure. (a1-3) Blank control mice. (b1-3) C. albicans vaginal infected mice. (C1-3) C. albicans vaginal infected mice with adenovirus vehicle control. (d1-3) C. albicans vaginal infected mice with lncRNA9708-1 overexpression (JPG 837 kb) [file 11046_2021_530_MOESM2_ESM.jpg]

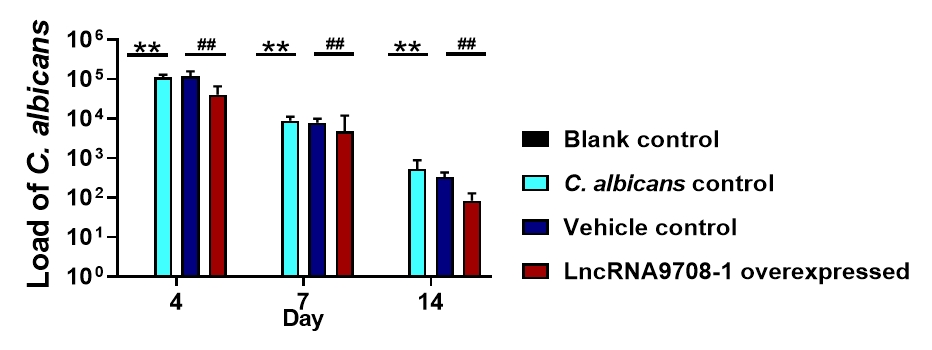

Supplement: Supplementary file 3 — Vaginal bacteria load after C. albicans implantation. C. albicans titers were determined by enumerating colony-forming units (CFU) in vaginal washes at 4,7, 14 day after infection. Results are meta data from three independent experiments, each with nine mice infection group (JPG 80 kb) [file 11046_2021_530_MOESM3_ESM.jpg]
